# Duplex Uterus and Bipartite Vagina

**Published:** 1914-06

**Authors:** 


**Duplex Uterus and Bipartite Vagina.** Geo. R. Smith of Mt. Vernon, N. Y., Med. Jour., May 16, 1914, reports a case in a 4-para, discovered during an attempt at prophylactic abortion. The right cervix was virginal.

